# Érythrodermie néonatale: ne pas méconnaitre un déficit immunitaire

**DOI:** 10.11604/pamj.2014.18.317.5182

**Published:** 2014-08-21

**Authors:** Aziza El Ouali, Yousra El Boussaadni, Fatima Ailal, Ahmed Aziz Bousfiha, Sihame Dikhaye, Noufissa Benajiba

**Affiliations:** 1 Service de Pédiatrie, CHU Mohammed VI, Université Mohamed I Oujda, Maroc; 2 Unité d'Immunologie Clinique Service des maladies infectieuses Pédiatriques, Hôpital Abderrahim Alharouchi CHU Casablanca, Maroc; 3 Service de Dermatologie CHU Mohammed VI, Université Mohamed I Oujda, Maroc

**Keywords:** Erythrodermie, nouveau né, syndrome d´Omenn, Erythroderma, newborn, Omenn syndrome

## Abstract

Les érythrodermies du nouveau-né sont des tableaux rares, d’évolution chronique et potentiellement mortelles. Leurs étiologies sont diverses et variées dominées par les ichtyoses, le déficit immunitaire primaire, ou plus exceptionnellement le psoriasis sévère, les maladies métaboliques, la dermatite atopique ou une cause infectieuse. La détermination rapide de la cause sous-jacente est cruciale pour une meilleure prise en charge. Cependant, le diagnostic constitue un défi pour le clinicien et est souvent retardé en raison de la nature non spécifique des signes cliniques et histopathologiques. L'objectif de notre travail est de rappeler aux praticiens une étiologie grave de l’érythrodermie néonatale: le déficit immunitaire ceci à travers l'observation d'un nourrisson suivi pour une érythrodermie évoluant depuis la naissance, hospitalisé pour une fièvre prolongée, et chez qui un interrogatoire minutieux, un examen clinique soigneux, ainsi que l’évolution ont pu redresser le diagnostic vers un déficit immunitaire.

## Introduction

Les érythrodermies du nouveau né, sont des entités rares [1]. Elles sont définies par un érythème généralisé d’évolution chronique touchant au moins 90% de la surface corporelle [2]. Les étiologies sont multiples; cutanées, génétiques, métaboliques, immunologiques et nutritionnelles [3]. La prise en charge thérapeutique doit être rapidement envisagée; tant sur le plan symptomatique visant à limiter les complications métaboliques, hydro-électrolytiques et infectieuses que sur le plan étiologique. Le pronostic dépend essentiellement du diagnostic mais également du terrain sous jacent [2].

## Patient et observation

Nous rapportons l'observation d'un garçon âgé de 2 mois, cadet d'une fratrie de 2, issu d'un mariage consanguin de 2éme degré et ayant comme antécédents un décès chez un frère à l’âge de 2 jours. La grossesse s'est déroulée normalement, menée à terme avec un accouchement par voie basse. Le poids de naissance était à 3Kg 300. L'anamnèse retrouvait la notion d'une érythrodermie évoluant depuis la naissance, traitée comme ichtyose. Le nourrisson a été admis pour une symptomatologie faite d'une fièvre prolongée, de diarrhée chronique, et de stagnation pondérale. L'examen clinique retrouvait un nourrisson fébrile à (38,2°C), hypotonique, pesant 3 kg200 (-3 DS) avec des œdèmes des membres inférieurs, des adénopathies axillaires et inguinales bilatérales ceci sans hépato-splénomégalie. L'examen clinique objectivait également une éruption érythémato- squameuse diffuse, et une alopécie. Le bilan biologique initial notait une hyperleucocytose, une hyper lymphocytose et une hyper éosinophilie. La CRP et la procalcitonine étaient élevées, le taux de protidémie était bas. La fonction rénale était normale. La radiographie thoracique était sans anomalie. Les prélèvements bactériologiques étaient négatifs hormis la ponction lombaire qui était en faveur d'une méningite lymphocytaire. Le diagnostic d'une infection bactérienne sévère a été évoqué; Le patient a été mis sous bi antibiothérapie à large spectre. L’évolution a été marquée par l'apparition d'autres foyers infectieux (otite purulente, une onychomycose et un muguet buccal). Devant ce tableau clinique, un déficit immunitaire était fortement suspecté. La sérologie HIV était négative. Le syndrome d'omenn a été évoqué en premier vu les données anamnestiques et clinico-biologiques. L'enfant a succombé à un choc septique avant la confirmation diagnostique.

## Discussion

Indépendamment de la cause, l’érythrodermie néonatale est une condition potentiellement mortelle qui doit être correctement reconnue par les médecins, les dermatologues et les pédiatres [4]. Cependant peu d’études à ce sujet sont retrouvées dans la littérature, contrairement aux adultes [5, 6]. Sur le plan clinique, elle se définit comme une éruption érythémateuse plus ou moins squameuse touchant plus de 90% de la surface corporelle [2, 7]. Ce qui était le cas pour notre malade. Différentes hypothèses diagnostiques peuvent être discutées. Ainsi une étiologie infectieuse notamment une nécrolyse épidermique staphylococcique SSSS ou une candidose congénitale ou néonatale doit être éliminée [8, 9]. Les ichtyoses simples (ichtyoses congénitales non bulleuses et ichtyoses lamellaires) ou complexes: syndrome de Netherton, la dermatite atopique, le psoriasis, et la dermite séborrhéique sont également incriminés [1–3]. Les érythrodermies métaboliques, extrêmement rares, sont généralement associées à des manifestations neurologiques aiguës et sévères [1]. Certains antibiotiques, en particulier, la ceftriaxone et la vancomycine peuvent être responsables d'un tableau d’érythrodermie chez le nouveau né [10]. Les déficits immunitaires congénitaux, notamment le syndrome d'Omenn,la réaction du greffon contre l'hôte ou autres déficits immunitaires combinés sévères (SCID), représentent une cause fréquente d’érythrodermie en période néonatale [8, 11, 12] et doivent être évoqués devant toute érythrodermie sévère ou résistante aux traitements topiques standards associée ou non à une organomégalie, un retard staturo-pondéral,une diarrhée chronique, des infections systémiques récidivantes et une alopécie sévère[1, 3]. Dans une étude rétrospective menée par Pruszkowski et al, le diagnostic de déficit immunitaire était retenu chez (30%) des patients ayant une érythrodermie néonatale [13]. La réalisation d'un bilan immunitaire s'avère alors nécessaire et repose sur l'hémogramme, l’électrophorèse des protides, le dosage pondéral des immunoglobulines et le phénotypage lymphocytaire [3, 12]. La biopsie cutanée permettra d'orienter le diagnostic en mettant en évidence un infiltrat inflammatoire important et une nécrose kératinocytaire [1].

Chez notre patient, l’âge du début de l’érythrodermie, la notion de décès dans la fratrie, la consanguinité parentale, et les signes cliniques extra cutanés associés, ont permis d'orienter le diagnostic vers un déficit immunitaire. La numération sanguine était en faveur d'une hyper éosinophilie, une hyperleucocytose, et d'une hyper lymphocytose. Toutefois en raison du manque du laboratoire spécifique et de l’évolution rapidement fatale, le diagnostic de déficit immunitaire n'a pas été confirmé. Quelle qu'en soit l’étiologie, l’érythrodermie justifie une prise en charge adaptée en milieu hospitalier; visant à optimiser la barrière cutanée et à guetter les complications aussi bien métaboliques, hydro électrolytiques et infectieuses (cutanées ou générales). Les déficits immunitaires doivent en outre être pris en charge par des équipes d'hématologie pédiatrique et justifient un traitement spécifique. Dans le syndrome d'Omenn et certains déficits immunitaires combinés sévères, la greffe de moelle a montré des résultats encourageants [1, 11].

## Conclusion

Les érythrodermies néonatales sont rares, mais peuvent être rapidement fatales. Elles restent un défi diagnostique pour les pédiatres, les dermatologues, les immunologistes et les généticiens. L'histoire familiale, le mode de début, les signes cliniques associés, l'existence d'infections récidivantes et d'un retard statural, doivent être recherchés pour ne pas passer à coté d'un déficit immunitaire ;Cette cause qui est soutenue par les données de la littérature, doit être exclue dans tous les cas d’érythrodermie néonatale même en l'absence de manifestation systémique, et faire penser à la réalisation d'un bilan immunitaire.
